# Subcutaneous Sweet Syndrome Successfully Treated With Ustekinumab in a Patient With Ulcerative Colitis

**DOI:** 10.14309/crj.0000000000000881

**Published:** 2022-11-22

**Authors:** Kelly A. Hu, Jeanne Shen, Kerri Rieger, Mike T. Wei, John Gubatan

**Affiliations:** 1 Department of Medicine, Stanford University School of Medicine, Stanford, CA; 2 Department of Pathology, Stanford University School of Medicine, Stanford, CA; 3 Department of Dermatology, Stanford University School of Medicine, Stanford, CA; 4 Division of Gastroenterology and Hepatology, Stanford University School of Medicine, Stanford, CA

## Abstract

Ustekinumab, an inhibitor of the interleukin-12/23 pathway, received Food and Drug Administration (FDA) approval in 2019 for induction and maintenance therapy in patients with moderate-to-severe ulcerative colitis (UC). Data regarding the efficacy of ustekinumab in the treatment of extraintestinal manifestations of UC are unclear. Sweet syndrome, an acute febrile neutrophilic dermatosis, is a cutaneous manifestation of inflammatory bowel disease that parallels disease activity. In this study, we present the first case of subcutaneous Sweet syndrome with sterile osteomyelitis in a patient with UC successfully treated with ustekinumab.

## INTRODUCTION

Extraintestinal manifestations (EIMs) of inflammatory bowel disease (IBD) are well established, with up to 40% of IBD cases complicated by EIMs; cutaneous EIMs are seen in over 10% of patients.^1^ Sweet syndrome (SS), or acute febrile neutrophilic dermatosis, is a reactive mucocutaneous manifestation of IBD. SS presents as erythematous papules/plaques usually involving the face/neck and limbs with associated systemic symptoms such as fever and arthromyalgias.^2^ Histologically, SS is characterized by dense neutrophilic infiltrate in the dermis.^1^ SS is more common in women in the third to fifth decades of life. Although usually associated with active IBD and reflecting intestinal disease activity, particularly colonic involvement, it can present before, after, or at the time of initial IBD diagnosis.^3–5^ There is limited evidence regarding the efficacy of ustekinumab in the management of EIMs of ulcerative colitis (UC), particularly SS.

## CASE REPORT

The patient is a 50-year-old woman with a medical history of left-sided UC (Montreal classification E2) since 2011. She previously failed several therapies including mesalamine, azathioprine, infliximab, adalimumab, and vedolizumab, as well as a fecal microbiota transplant trial. Although briefly lost to follow-up, she represented with worsening abdominal pain, diarrhea, hematochezia, and progressive acute-on-chronic bilateral lower extremity pain.

**Figure 1.**
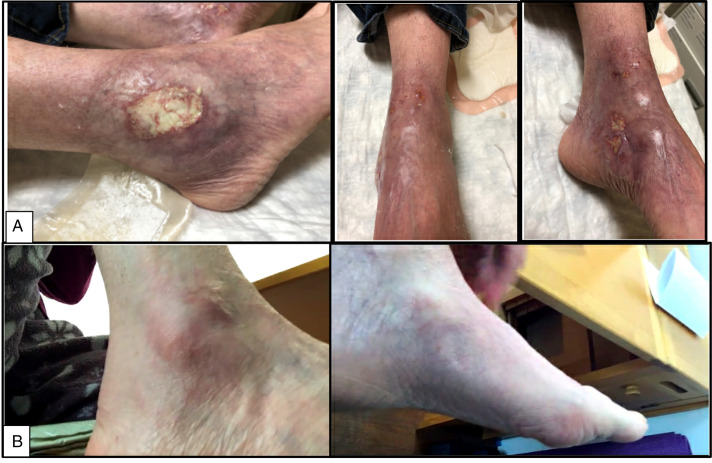
(A) Right lateral malleolus with 2.5-cm shallow erosion with slightly violaceous borders and central bright yellow fibrinous debris. There was slight atrophy of the surrounding skin with mild edema and hyperpigmentation (left image). Left medial malleolus/dorsal foot with grouped irregular shallow ulcers with surrounding mild violaceous erythema (middle image). Right flank with linear surgical ulcer with surrounding erythema and induration (right image). (B) Resolution of erosions and ulcerations after treatment.

**Figure 2.**
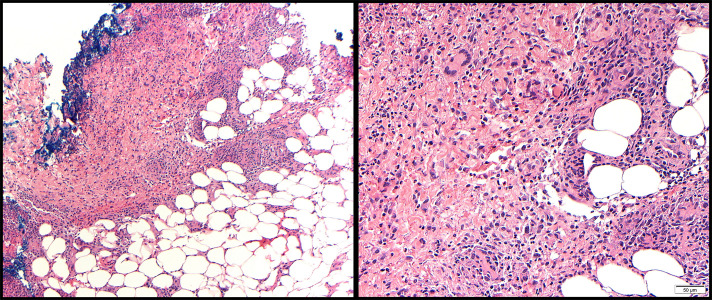
Left ankle biopsies showed mixed septal and lobular panniculitis with granulomatous and neutrophilic inflammation. Histologic sections show a mildly spongiotic epidermis with dense granulomatous and neutrophilic inflammatory infiltrate in the deep dermis and subcutis (left image). The inflammatory infiltrate, which consists of prominent neutrophils, histiocytes, and scattered lymphocytes, surrounds vascular and adnexal structures, as well as adipocyte lobules. Definitive features of vasculitis are not seen. Periodic acid-Schiff diastase, Grocott methenamine silver, Gram, and Fite staining fail to highlight microorganisms (right image). These findings, combined with the clinical appearance of numerous intact, nonulcerated lesions and absence of an undermined border of the ulcerated lesions, support a diagnosis of SS rather than a related neutrophilic dermatosis such as pyoderma gangrenosum. SS, Sweet syndrome.

On admission, she was febrile to 38.2°C and tachycardic. Examination was notable for tender subcutaneous nodules on the bilateral ankles with violaceous erythema, ulceration, and hemorrhagic bullae (Figure 1). Laboratory test results were significant for erythrocyte sedimentation rate 120 mm/hr, C-reactive protein 32 mg/dL, fecal calprotectin 1,655 μg/g, and negative *Clostridioides difficile* as well as thrombocytosis and borderline leukocytosis (neutrophilic predominance). The Mayo Severity Index was 11. Magnetic resonance imaging of the bilateral lower extremities was concerning for multifocal osteomyelitis with adjacent abscess of bilateral ankles. Bone and skin biopsies were obtained for histology/culture. Bone biopsy Gram stain and cultures were negative. Pathology demonstrated focal neutrophilic inflammation of the bone consistent with sterile osteomyelitis in the setting of systemic inflammation. Left ankle skin biopsies revealed a spongiotic epidermis with dense granulomatous and neutrophilic inflammatory infiltrate in the deep dermis/subcutis without evidence of microorganisms. The histopathologic and clinical findings were consistent with subcutaneous SS (Figure 2). The patient was discharged with prednisone for UC flare and SS with some improvement in her cutaneous and gastrointestinal symptoms.

**Figure 3.**
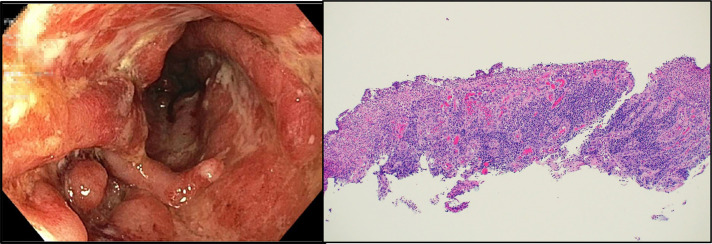
Colonoscopy demonstrated severe (Mayo Endoscopy Score 3) proctosigmoid ulcerative colitis to 30 cm (left image). Sigmoid biopsies showed significant immune cell infiltration with crypt architectural distortion concerning for severe chronic active colitis. No evidence of granulomas, dysplasia, or cytomegalovirus (right image).

A colonoscopy at discharge demonstrated severe (Mayo Endoscopy Score 3) proctosigmoid UC to 30 cm. Sigmoid colon biopsies showed severe chronic active colitis (Figure 3). Because she had failed several immunomodulators and biologic therapies with ongoing colitis and concurrent cutaneous symptoms of SS, she was started on ustekinumab. One month later, the patient's cutaneous lesions were improving off prednisone (Figure 1). She had repeat magnetic resonance imaging, which demonstrated resolution of the sterile osteomyelitis and abscess. Four months after starting ustekinumab, she was seen in the gastroenterology clinic for follow-up and reported complete resolution of her cutaneous SS off steroids and improving UC symptoms (less frequent and more formed stools, minimal hematochezia). Fecal calprotectin improved to 150 μg/g, and the Mayo Severity Index was 4. Colonoscopy repeated at 8 months demonstrated moderate sigmoid inflammation (Mayo Endoscopy Score 2, improved from 3).

## DISCUSSION

Our case highlights the novel use of ustekinumab in the treatment of subcutaneous SS with sterile osteomyelitis in a patient with flaring UC. SS is a neutrophilic dermatosis related to pyoderma gangrenosum and erythema nodosa, both of which are other cutaneous EIMs that can be seen in IBD.^5^ It was first reported in conjunction with IBD in 1988, specifically in relation to Crohn's disease (CD); since then, IBD has been found to be the third most common disease associated with SS, more often seen in relation to CD than UC.^1,5,6^ SS has many triggers including pregnancy and malignancy; in the case of this patient, it was likely provoked by her underlying IBD.^2^ Interestingly, there have been case reports of IBD-related SS induced by azathioprine therapy, which this patient had received in the past.^3^ However, azathioprine was unlikely the trigger for SS in our patient because she had been off this medication for several years.

There is no consensus on the treatment of SS in IBD, and similar patients have been treated with a variety of immunosuppressives, including topical/intravenous corticosteroids, infliximab, and golimumab.^7–11^ Ustekinumab has been approved for the treatment of CD since 2016 and just recently received regulatory approval for the treatment of UC in 2019.^12,13^ The data on the efficacy of ustekinumab for the treatment of EIMs of IBD and particularly SS are limited and mixed. A post hoc analysis by Narula et al of the UNITI studies that led to regulatory approval of ustekinumab for CD found that the drug had no significant effect on EIMs in CD; SS was not included in the analysis, but other neutrophilic dermatoses such as pyoderma gangrenosum and erythema nodosum were assessed.^14^ Guillo et al conducted a separate systematic literature review and found ustekinumab to be a particularly effective treatment for the dermatologic and rheumatologic EIMs of IBD; although again, SS was not included in the analysis.^15^ In 2021, de Risi-Pugliese et al conducted a multicenter retrospective study evaluating response to ustekinumab in 7 patients with CD with concurrent neutrophilic dermatosis. In this study, ustekinumab induced neutrophilic dermatosis remission in 6 of 7 cases (86%), complete response in 4 of 7 cases (57%), partial response in 2 of 7 cases (29%), and no response in 1 case.^16^ To date, there have been no prior reports of use of ustekinumab in patients with UC and concurrent neutrophilic dermatoses.

In conclusion, we present the first case of UC complicated by subcutaneous SS and sterile osteomyelitis successfully treated with ustekinumab. SS is a neutrophilic dermatosis that is a rare EIM of IBD classically characterized by tender cutaneous lesions, although, as in the case of our patient, can also have uncommon presentations such as multifocal sterile osteomyelitis.^17^ There is limited evidence supporting the use of ustekinumab in treating EIMs of CD and a lack of data regarding its efficacy in treating EIMs of UC. Our case fills a major gap in the literature and highlights the clinical efficacy of ustekinumab for SS and UC, especially in patients who have a contraindication to, failed previous treatment with, or otherwise cannot tolerate corticosteroids and tumor necrosis factor-α antagonists.

## DISCLOSURES

Author contributions: KA Hu reviewed the literature, prepared the case report, and wrote the manuscript. MT Wei provided critical feedback and helped draft the manuscript. J. Shen provided the GI pathology interpretation and images. K. Rieger provided the dermatopathology interpretation and images. J. Gubatan obtained the endoscopy and pathology images, provided critical feedback, revised the manuscript, and is the article guarantor.

Acknowledgments: J.G. is in part supported by a Chan Zuckerberg Biohub Physician Scientist Scholar Award, a NIH NIDDK LRP Award (L30 DK126220), and a Doris Duke Physician Scientist Fellowship Award (Grant #2021091).

Financial disclosure: MT Wei is a consultant for Neptune Medical and AgilTx.

Informed consent was obtained for this case report.
